# Comparative analysis of deep learning architectures for breast region segmentation with a novel breast boundary proposal

**DOI:** 10.1038/s41598-025-92863-3

**Published:** 2025-03-14

**Authors:** Sam Narimani, Solveig Roth Hoff, Kathinka Dæhli Kurz, Kjell-Inge Gjesdal, Jürgen Geisler, Endre Grøvik

**Affiliations:** 1 Department of Physics, Norwegian University of Science and Technology, Trondheim, Norway; 2 Research and Development Department, More og Romsdal Hospital Trust, Aalesund, Norway; 3 Department of Radiology, More og Romsdal Hospital Trust, Aalesund, Norway; 4 Department of Circulation and Medical Imaging, Norwegian University of Science and Technology, Trondheim, Norway; 5 Department of Electrical Engineering and Computer Science, The University of Stavanger, Stavanger, Norway; 6 Department of Radiology, Stavanger University Hospital, Stavanger, Norway; 7 Department of Diagnostic Imaging, Akershus University Hospital, Lorenskog, Norway; 8 NordicCAD AS, Aalesund, Norway; 9 Institute of Clinical Medicine, University of Oslo, Lorenskog, Norway; 10 Department of Oncology, Akershus University Hospital, Lorenskog, Norway

**Keywords:** Breast region, DCE-MRI, Deep learning methods, Segmentation, Breast cancer, Risk factors, Biomedical engineering

## Abstract

Segmentation of the breast region in dynamic contrast-enhanced magnetic resonance imaging (DCE-MRI) is essential for the automatic measurement of breast density and the quantitative analysis of imaging findings. This study aims to compare various deep learning methods to enhance whole breast segmentation and reduce computational costs as well as environmental effect for future research. We collected fifty-nine DCE-MRI scans from Stavanger University Hospital and, after preprocessing, analyzed fifty-eight scans. The preprocessing steps involved standardizing imaging protocols and resampling slices to ensure consistent volume across all patients. Using our novel approach, we defined new breast boundaries and generated corresponding segmentation masks. We evaluated seven deep learning models for segmentation namely UNet, UNet++, DenseNet, FCNResNet50, FCNResNet101, DeepLabv3ResNet50, and DeepLabv3ResNet101. To ensure robust model validation, we employed 10-fold cross-validation, dividing the dataset into ten subsets, training on nine, and validating on the remaining one, rotating this process to use all subsets for validation. The models demonstrated significant potential across multiple metrics. UNet++ achieved the highest performance in Dice score, while UNet excelled in validation and generalizability. FCNResNet50, notable for its lower carbon footprint and reasonable inference time, emerged as a robust model following UNet++. In boundary detection, both UNet and UNet++ outperformed other models, with DeepLabv3ResNet also delivering competitive results.

## Introduction

Whole breast segmentation is a pivotal step in assessing the risk of breast cancer^1^. Accurate breast region segmentation can be beneficial not only for lowering computational cost for predicting breast cancer but also to focus on quantitative analysis of breast cancer^2,3^. Among all medical imaging modalities, MRI plays a significant role in high quality visualization of the whole breast^4^. However, interpretation of breast MRI can be challenging due to noise and artifacts from surrounding anatomical structures^5^.

In recent years, deep learning (DL) techniques have emerged as powerful tools in medical image analysis, offering the potential to automate and improve various tasks such as segmentation^6–8^. Segmentation, the process of partitioning an image into multiple regions or segments, is a fundamental step in medical image analysis. In fact, it enables a fast and automatic delineation of Region of Interest (ROI), such as tumors or anatomical regions, with high precision and accuracy^9^. Segmentation of the breast region in MRI images makes it possible to create automatic models for breast density measurement^10^. This process not only enhances the efficiency of data processing but also contributes to the rapid training and analysis of AI models, promoting more environmentally sustainable data processing. Furthermore, precise segmentation of the breast region facilitates the localization and characterization of abnormalities, thereby assisting radiologists in their diagnostic decision-making process^11^.

In the last decade, significant advancements have been made in breast region segmentation due to the development of numerous AI architectures^12–14^. These advancements mark a notable shift in segmentation techniques, transitioning from traditional feature-based machine learning methods, such as clustering^15,16^, to more advanced deep learning approaches, including UNet and its variants^17^. These efforts have led to improved diagnostics and more precise stratification of breast cancer tumors^18^. Despite these advances, no comparative study has been conducted to evaluate the performance of well-known DL methods for breast region segmentation. Therefore, our study aims to fill this gap by comparing seven prominent DL architectures for segmenting breast regions in DCE-MRI. The goal is to identify the most competitive network that minimizes computational costs while effectively eliminating background noise.

## Materials and methods

### Data

#### Data description

The dataset utilized in this study consists of DCE-MRI scans obtained from 59 patients at Stavanger University Hospital in 2008. The DCE sequence comprises one pre- and five post-contrast image series with a temporal resolution of 63 seconds. Table 1 provides a detailed description of the dataset and screening parameters.

#### Image acquisition

All DCE-MRI scans were acquired using a 1.5 Tesla MRI scanner, Philips Intera, with a dedicated breast coil equipped with SENSE technology for high-quality and high-resolution images. Imaging parameters included T1 weighted fast spoiled gradient echo (FSPGR) sequence, with a scan resolution of 0.9659 x 0.9659 mm^2^ and dynamic acquisition time of 6 minutes and 20 seconds following contrast agent administration.

**Table 1:** Detailed Specifications and Imaging Features of MRI Scans.

Category	Attribute	Description
Study Information	Patient number	59
	Weight (kg)	70.6 ${\pm }$ 8.4
	Patient Position	Head First Prone (HFP)
	Number of Images per Patient	6 (1 pre-contrast, 5 post-contrast)
Scanner Properties	Scanner Model	Philips Intera MRI Scanner
	Magnetic Field Strength (T)	1.5
	Coil Technology	SENSE Technology
Image characteristics	Image Dimensions	(352,352,150), (352,352,140), (352,352,120)
	Pixel Spacing (mm)	0.9659 x 0.9659
	Slice Thickness (mm)	2
	Field of View (FOV) (mm)	340
Imaging Features	MRI Sequence	T1 weighted fast spoiled gradient echo (FSPGR)
	Repetition Time (TR)	6.91
	Echo Time (TE)	3.39
	Flip Angle	12

### Preprocessing

The initial dataset, comprised of imaging data in the DICOM standard format, underwent a meticulous cleansing process to ensure data integrity. Subsequently, both pre- and post-contrast images were automatically identified and converted to the NIFTI format, a prerequisite for our modeling endeavors. To ensure consistent data volume, a random oversampling method was applied to minority volumes. This approach simplifies data preparation, making it easier to process before feeding it into the model for further analysis. In addition, breast regions were annotated in detail, adhering to predefined boundary criteria outlined in breast boundary assumptions thereby providing insights for subsequent analyses.

### Deep learning networks

Over the past few decades, numerous segmentation models have been introduced by researchers. Among these, encoder-decoder based models with skip connections have garnered significant attention due to their effectiveness in retaining important features during training^19^. In this study, we employed seven widely recognized segmentation architectures-UNet, UNet++, DenseNet, FCNResNet50, FCNResNet101, DeepLabv3ResNet50, and DeepLabv3ResNet101-to train on our dataset. These models were selected for their proven efficacy in medical image segmentation tasks^19–24^.

UNet, introduced by Ronneberger et al. in 2015^19^, is one of the most popular segmentation methods. It consists of contraction and expansion pathways connected by skip connections. These skip connections help the model retain important features that might otherwise be forgotten during the training process. UNet++ is an improved version of UNet, designed to achieve superior results. In UNet++, the skip connections were redesigned to reduce the loss of important features between the contraction and expansion pathways, enhancing the overall performance of the model^20^. DenseNet, another architecture utilized in this study, has demonstrated promise in propagating features throughout the model. In DenseNet, every layer is connected to other layers, thereby enhancing feature propagation across the entire network and improving the model’s ability to learn complex patterns^21^. Given that DenseNet is primarily used for classification tasks, we employed its feature extraction part along with a decoder, excluding skip connections, to examine the impact of their absence in a deeper model. Next network is FCNResNet comprising a ResNet as the feature extractor and an FCN header^22^ for upsampling or decoding. ResNet’s structure, which includes residual blocks, has proven effective^23^, while the FCN header connects to each feature level, serving as a skip connection. Last architecture, DeepLabv3 is renowned for its Atrous Spatial Pyramid Pooling (ASPP) block^24^. Following the ResNet feature extractor, ASPP is applied and subsequently added to the decoder part of the architecture for upsampling. Table 2 provides practical information about the networks, including learning parameters, the number of layers, and their distinctive features. This comparative analysis offers valuable insights into the strengths and applications of each model in medical image segmentation tasks.

**Table 2:** Model specification and features.

Architecture name	Layers	Learning Parameters	Special Features
UNet	141	31,112,641	Simple skip connection
UNet++	240	9,119,044	Dense skip connection
DenseNet	1216	70,536,843	Reusing Feature-maps in subsequent blocks
FCNResNet50	157	32,943,617	Strong feature extractor alongside FCN header
FCNResNet101	293	51,935,745	
DeepLabv3ResNet50	184	39,630,593	Atrous Spatial Pyramid Pooling (ASPP)
DeepLabv3ResNet101	320	58,622,721	

### Evaluation

In the evaluation section, we assess the performance of our models using the Dice loss function^25^ and k-fold cross-validation^26^. The Dice loss function, a promising evaluator for measuring overlap in segmentation tasks, ensures precise model predictions. We employed 10-fold cross-validation, partitioning the dataset into 10 equal parts to validate the model’s consistency and performance across different data subsets. The Dice loss function is calculated as shown in relation 1, where $P$ and $G$ refer to predicted and ground truth labels, respectively.

1 $$\begin{aligned} \text {Dice loss}(P,G) = 1 - 2 \cdot \frac{ P \cap G}{P + G} \end{aligned}$$

Other evaluation metrics used are Intersection over Union (IoU), Precision, and Recall, defined in relations 2 to 4, respectively.

2 $$\begin{aligned} \text {IoU}&= \frac{ TP}{ TP + FN + FP} \end{aligned}$$

3 $$\begin{aligned} \text {Precision}&= \frac{TP}{TP + FP} \end{aligned}$$

4 $$\begin{aligned} \text {Recall}&= \frac{TP}{TP + FN} \end{aligned}$$

Where $TP$, $FP$, and $FN$ are true positives, false positives, and false negatives, respectively.

On the other hand, the carbon footprint is a critical factor in AI applications that must be considered^27^. The average carbon footprint for producing 1 kWh of energy is reported to be 475 grCO2^28^. Consequently, the carbon footprint for each fold can be calculated using relation 5:

5 $$\begin{aligned} \text {CFP} = \frac{0.475 \cdot \text {TT}}{3600} \end{aligned}$$

where $\text {CFP}$ represents the carbon footprint in kilograms of CO2 for each fold, and $\text {TT}$ is the training time in seconds.

### Breast boundaries

Accurately annotating the boundaries of the breast region has been a persistent challenge, as highlighted in previous studies^29^. This challenge arises from the similar intensities observed in imaging for the upper chest wall, fibroglandular tissue, and pectoral muscles, making differentiation difficult^30^.

To improve breast region segmentation, we propose a novel boundary framework that not only captures the anatomical structure of the breast but also accommodates all breast cancer types. The anterior boundary is defined at the skin line to exclude low-intensity and noisy pixels, while the posterior boundary is aligned with the lungs to remove low-intensity and noisy pixels located dorsal to the chest wall. This framework ensures the exclusion of high-intensity pixels, such as those from blood vessels near the heart, which are often misclassified by models. Additionally, our method incorporates the pectoralis and intercostal muscles, as well as the ribs, to provide accurate staging for tumors invading the chest wall. Finally, removing noisy and low-intensity background anterior to the skin line creates a more balanced image, facilitating future approaches such as breast density assessment and lesion segmentation.

**Fig. 1:**
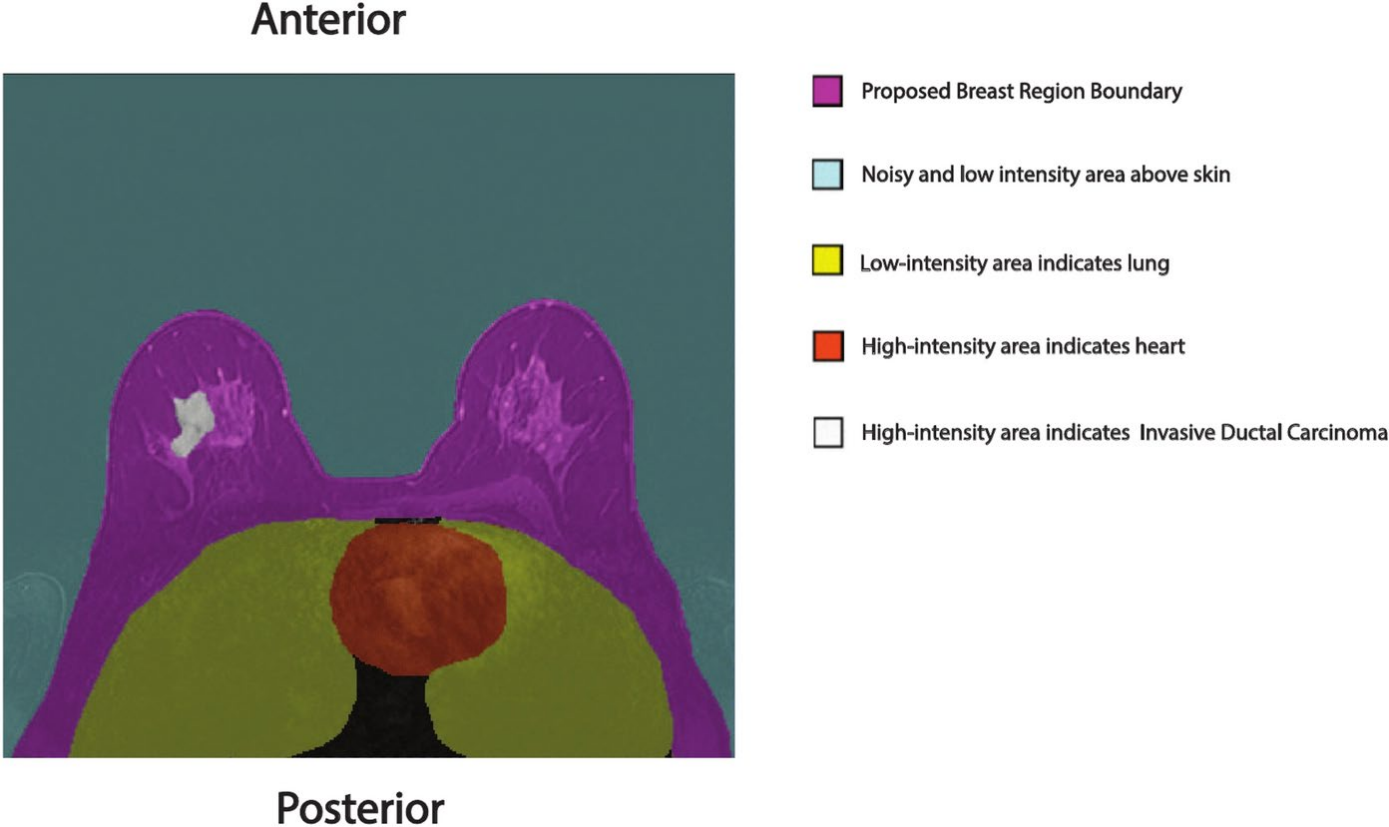
Illustration of distinct regions, highlighting the delineation of the proposed breast boundary.

Figure 1 illustrates the various regions of interest, highlighting low-intensity areas such as the background and lungs, and high-intensity areas like the heart and lesions, along with the delineated boundaries of the proposed breast region.

**Fig. 2:**
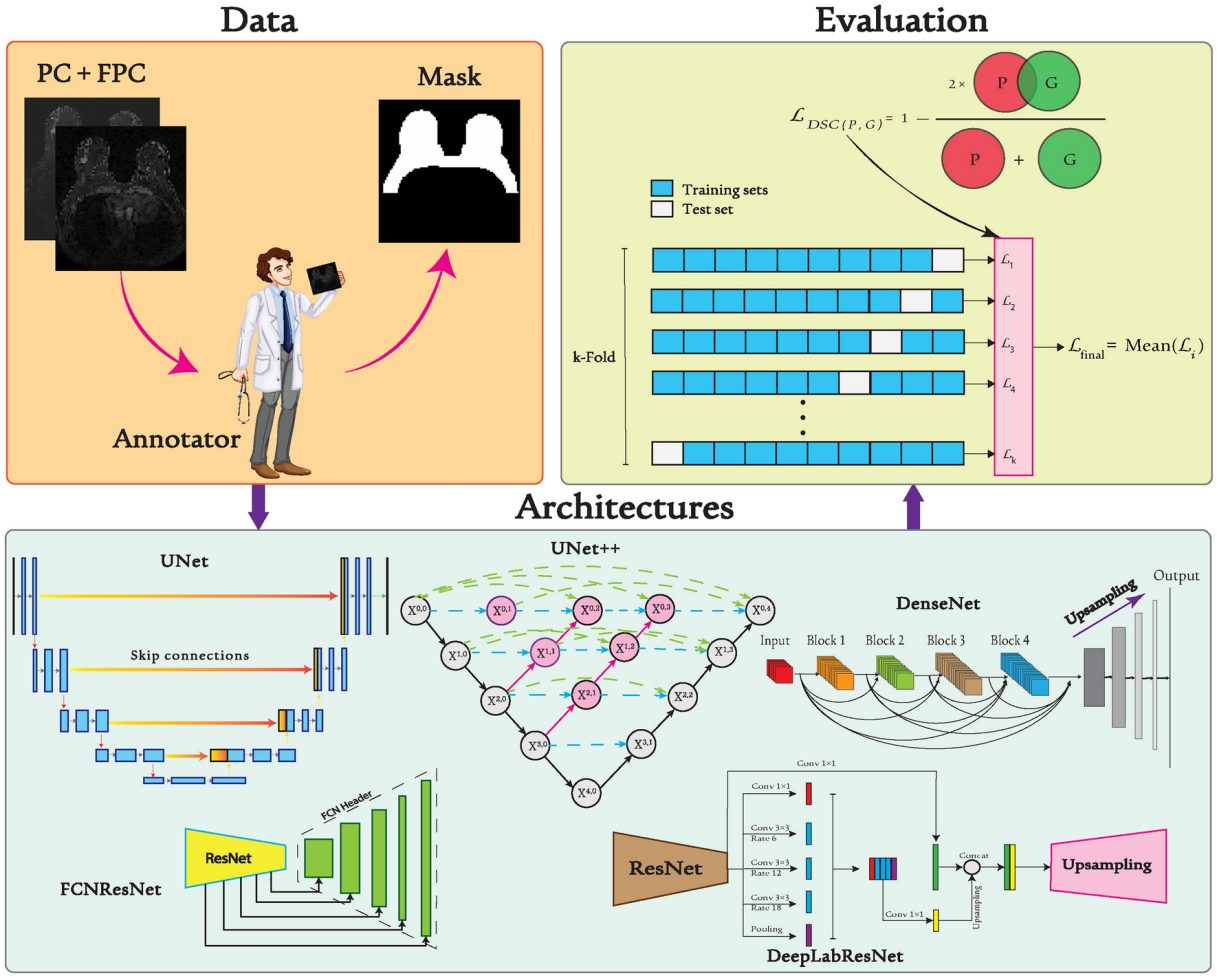
Schematic diagram of the study pipeline. Data part depicts data inputs (Pre-Contrast (PC) and First Post Contrast (FPC)), annotator and mask file, and the Architecture block consisting of seven distinct models, each trained individually. Evaluation methods, including 10-fold cross-validation and the Dice loss function, are also illustrated. (Annotator components adapted from Vecteezy.com)

### Setup

The simulations were conducted on a high-performance computing setup to ensure the efficient training and evaluation of the deep learning models. The hardware configuration utilized in this study includes an AMD Ryzen 9 7900X 12-Core Processor CPU, 32 GB of RAM, and an NVIDIA GeForce RTX 4090 GPU with 24 GB of GDDR6X memory. The power consumption of the GPU is reported to be 450 W by NVIDIA^31^, and the entire system consumes roughly 1 kWh of energy during each simulation.

Figure 2 depicts different pathways in data processing, the representation of various architectures, and the evaluation metrics and methods employed in our approach.

## Results

### Experiments

The input data consisted of both pre- and first post-contrast images, with corresponding masks serving as the ground truth outputs.The training process was conducted on a slice-by-slice basis and ,therefore, NIFTI images were fed to the model one slice at a time, as the training approach utilized a two-dimensional framework. Training involved various deep learning models using 10-fold cross-validation by utilizing Dice loss function. RAdam optimizer with an initial learning rate of 0.0001 in conjunction with a ReduceLROnPlateau scheduler was utilized to enhance model convergence and performance. This scheduler dynamically adjusted the learning rate based on validation performance metrics, aiming not only to minimize the Dice loss function, but also to accelerate training performance. Across all models, a consistent batch size of 8 was employed during training, with data shuffled to ensure robust model learning. Finally, a test subset comprising data from two patients was randomly partitioned to evaluate the model’s performance on previously unseen data.

### Model performance and generalizability

Table 3 displays Dice training and validation losses across different deep learning architectures at their best epochs. UNet++ achieves the lowest training loss of 0.0112 ${\pm }$ 0.0022, while FCN with ResNet50 also performs well with a Dice training loss of 0.0126 ${\pm }$ 0.0028. On the other hand, UNet architecture stands out for its superior validation results, indicating strong generalization to unseen data essential for real-world applications with validation loss of 0.0448 ${\pm }$ 0.0077. Following closely, UNet++ demonstrates competitive validation performance with losses of 0.0466 ${\pm }$ 0.0167, emphasizing its balanced model performance and generalizability. In contrast, DenseNet exhibits some of the poorest performance metrics, both in terms of training and validation loss, despite its deeper architecture. On the other hand, DeepLabv3 with a ResNet101 backbone achieves superior validation loss, second only to UNet.

**Table 3:** Training and validation Dice loss for various DL models for 10-fold cross-validation.

Models	Dice Training Loss	Dice Validation Loss
UNet	0.0146 ± 0.0024	0.0448 ± 0.0077
UNet++	0.0112 ± 0.0022	0.0466 ± 0.0167
DenseNet	0.0163 ± 0.0038	0.0525 ± 0.0082
FCNResNet50	0.0126 ± 0.0028	0.0474 ± 0.0100
FCNResNet101	0.0134 ± 0.0043	0.0497 ± 0.0067
DeepLabv3ResNet50	0.0140 ± 0.0036	0.0469 ± 0.0085
DeepLabv3ResNet101	0.0131 ± 0.0018	0.0462 ± 0.0034

**Fig. 3:**
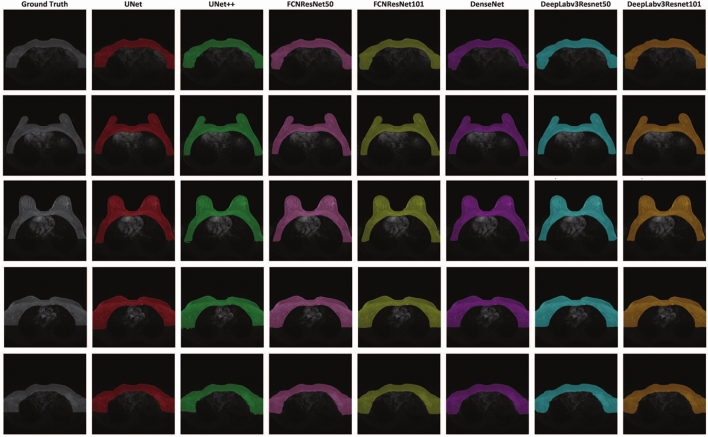
Model segmentation results across selected slices (from top to down row: first, 30^th^, middle, 120^th^ and last slices)

Figure 3 illustrates the segmentation results of different models across selected slices, specifically the first, 30th, middle, 120th, and last slices. These slices were chosen to represent the progression from the initial to the final slices of the volume, allowing for a comprehensive comparison of each model’s ability to maintain accuracy throughout the entire dataset.

### Internal breast segmentation and boundary detection

Table 4 provides a detailed comparison of model performance on the test dataset, evaluated using the Dice score, IoU, Precision, and Recall metrics. The UNet model achieved the highest performance across most metrics, except for Precision, where the DeepLabv3ResNet50 model outperformed others with a score of 0.9743 ${\pm }$ 0.0236. In contrast, the DenseNet model demonstrated the lowest performance overall, with a Dice score of 0.9641 ${\pm }$ 0.0183, an IoU of 0.9314 ${\pm }$ 0.0330, and a Precision of 0.9658 ${\pm }$ 0.0308.

**Table 4:** Performance metrics for various DL models during 10-fold cross-validation on test dataset.

Models	Dice	IoU	Precision	Recall
UNet	**0.9831 ± 0.0142**	**0.9671 ± 0.0264**	0.9707 ± 0.0277	**0.9963 ± 0.0069**
UNet++	0.9771 ± 0.0133	0.9556 ± 0.0245	0.9682 ± 0.0243	0.9867 ± 0.0142
DenseNet	0.9641 ± 0.0183	0.9314 ± 0.0330	0.9636 ± 0.0262	0.9658 ± 0.0308
FCNResNet50	0.9672 ± 0.0182	0.9370 ± 0.0330	0.9636 ± 0.0280	0.9720 ± 0.0312
FCNResNet101	0.9672 ± 0.0161	0.9369 ± 0.0295	0.9642 ± 0.0266	0.9712 ± 0.0263
DeepLabv3ResNet50	0.9721 ± 0.0188	0.9463 ± 0.0344	**0.9743 ± 0.0236**	0.9707 ± 0.0284
DeepLabv3ResNet101	0.9709 ± 0.0190	0.9441 ± 0.0347	0.9692 ± 0.0258	0.9735 ± 0.0286

**Fig. 4:**
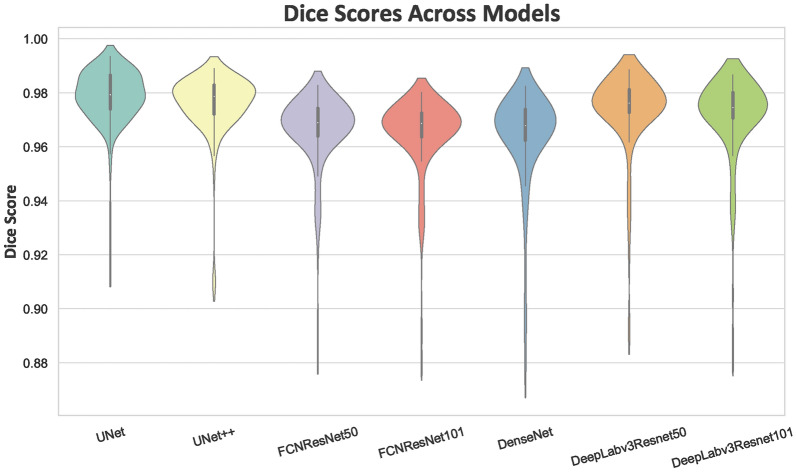
Dice score for different DL models on test subset

To provide a better understanding of the Dice score distribution,Figure 4 shows detailed distribution of Dice scores for each model on the test dataset, focusing on the median and range of performance. Notably, the UNet model has a median Dice score of 0.98, with a range from 0.91 to 0.995, highlighting its strong performance. Similarly, UNet++ achieves a median score of 0.98, with a range from 0.90 to 0.99. Close behind, DeepLabv3 with ResNet101 records a median of 0.975 with a slightly wider range from 0.88 to 0.99. On the other hand, the FCN models with ResNet50 and ResNet101 backbones show median Dice scores of 0.970 and 0.972, respectively, with ranges approximately from 0.88 to 0.985 for both. In contrast, DenseNet, though having a similar median Dice score of 0.970, shows the widest range of 0.87 to 0.99, indicating more variability and less consistent segmentation accuracy.

**Fig. 5:**
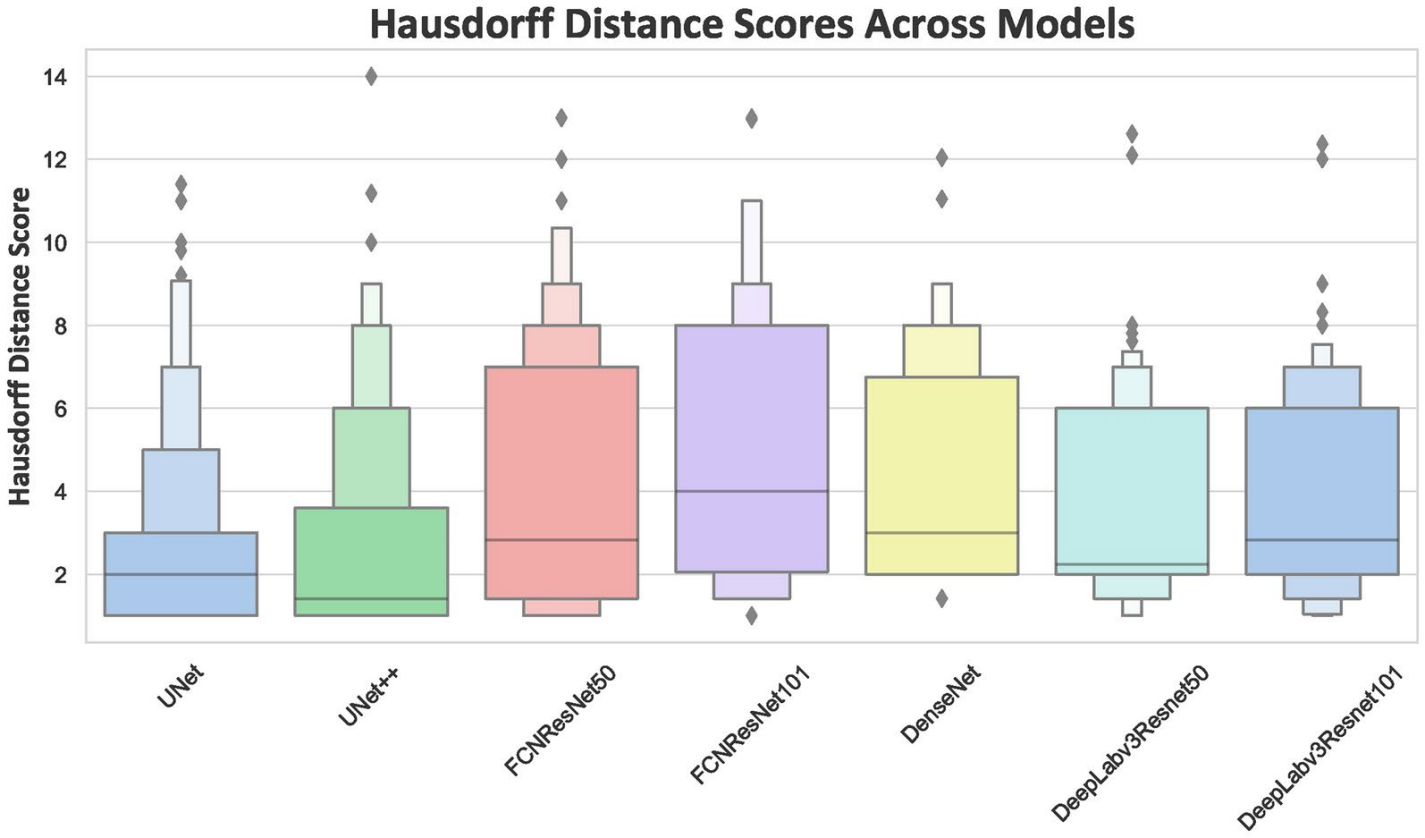
Hausdorff distance comparison across different models

As was evident in Figure 3, the segmentation of the breast region was generally excellent across all models, but differences in boundary detection were observed. To further evaluate and compare the boundary detection capabilities of each model, Figure 5 presents the Hausdorff distance on the test subset. As shown in the figure, UNet and UNet++ exhibit the lowest median Hausdorff distances, indicating their superior ability to accurately capture boundary details with minimal deviation. In contrast, the FCN models, particularly with ResNet50 and ResNet101 backbones, display higher median Hausdorff distances and a broader spread, highlighting greater variability in boundary detection and less precise segmentation at the edges. Similarly, DenseNet shows a wide range of Hausdorff distances, with a higher number of outliers, indicating that while it may perform adequately in some cases, it struggles with boundary accuracy in others. On the other hand, DeepLabv3 with ResNet50 and ResNet101 demonstrate relatively lower median Hausdorff distances compared to FCN models, but with a few outliers, suggesting these models are generally reliable but may still occasionally falter in capturing fine boundary details.

### Training and inference time

Training and inference time are critical considerations in efficiency and cost of the modelling. Table 5 Demonstrates training time results of 10-fold, with mean and standard deviation, alongside average inference time in the test set for diverse architectures. As presented in the table, FCN with ResNet50 shows the shortest training time at 87 ${\pm }$ 18 minutes, while DenseNet exhibits the longest at 185 ${\pm }$ 56 minutes. In terms of inference time per slice, UNet performs the best with 126 milliseconds, whereas DenseNet requires significantly more time at 696 milliseconds, highlighting varying computational efficiencies across these models. Despite possessing fewer trained parameters, UNet++ required more time, 199 ${\pm }$ 21, to train each fold. On the other hand, DeepLabv3ResNet50 demonstrated superior performance, followed by FCNResNet50, with training times of 104 ${\pm }$ 22 minutes per fold.

**Table 5:** Training and inference times across different models.

Models	Training time per fold (min)	Inference time per slice (msec)
UNet	136 ± 22	126
UNet++	199 ± 21	152
DenseNet	185 ± 56	696
FCNResNet50	87 ± 18	140
FCNResNet101	149 ± 35	266
DeepLabv3ResNet50	104 ± 22	161
DeepLabv3ResNet101	178 ± 37	294

**Fig. 6:**
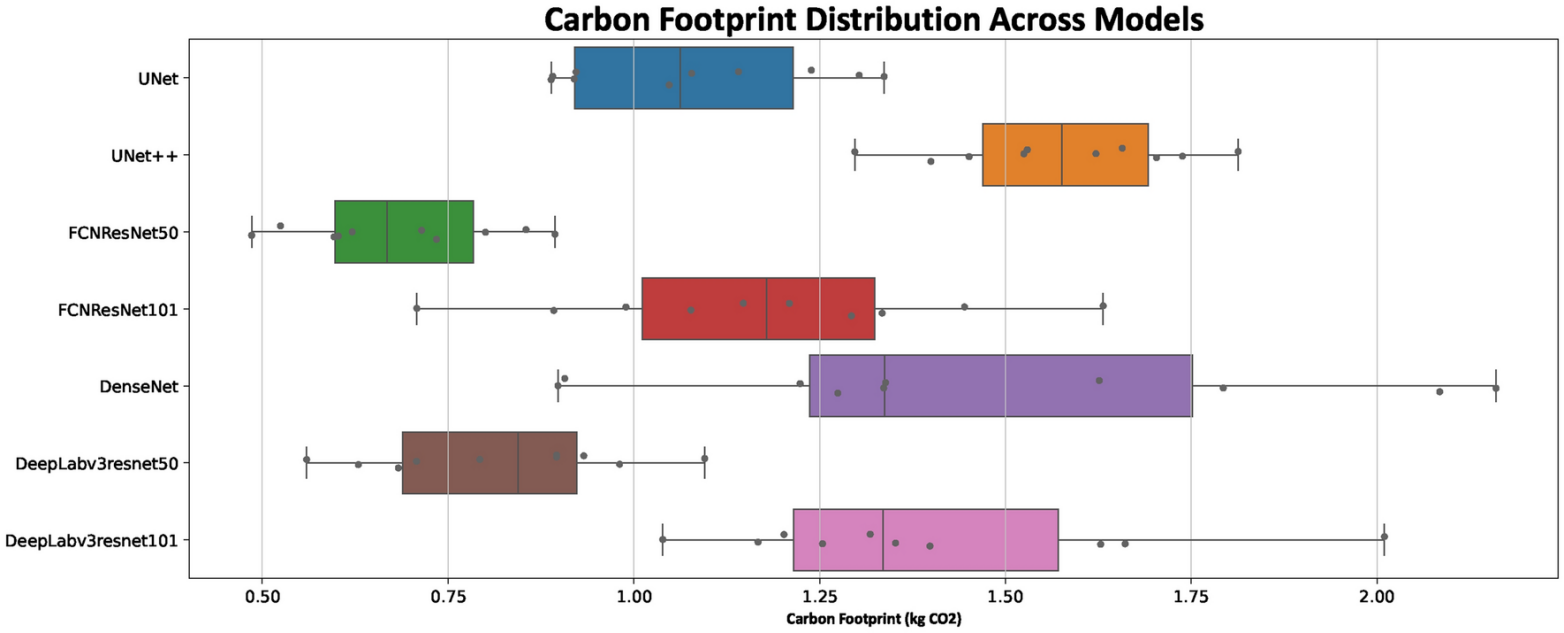
Carbon footprint across folds for various architectures

Recent research underscores the growing importance of assessing the carbon footprint as a key factor in evaluating environmental sustainability across various models^27,32,33^. Consequently, it is essential to investigate the carbon footprint associated with different network architectures during 10-fold cross-validation training. As illustrated in Figure 6, FCNResNet50 emerges as the most favorable and sustainable model, exhibiting a carbon footprint range of 0.45 to 0.85 kg CO2. This indicates that FCNResNet50 demonstrates the lowest environmental impact among the models analyzed. In contrast, DenseNet training is associated with higher energy consumption and, consequently, a larger carbon footprint. Following FCNResNet50, Deeplabv3ResNet50 displays the second-lowest carbon footprint, ranging from 0.55 to 1.15 kg CO2. Other models exhibit carbon footprints situated between these two extremes, reflecting a spectrum of environmental impacts.

## Discussion

This study offers a comprehensive evaluation of various state-of-the-art segmentation architectures applied to whole breast segmentation. The models examined include UNet, UNet++, DenseNet, FCNResNet50, FCNResNet101, DeepLabv3ResNet50, and DeepLabv3ResNet101. The results indicate significant differences in performance and training efficiency across these architectures, providing valuable insights into MR breast image analysis.

As outlined in Table 3, UNet exhibited a higher Dice loss function during training compared to UNet++. This may be attributed to UNet’s simpler architecture, which could enable more effective generalization during validation. Conversely, UNet++ introduces added complexity through its nested and dense skip connections, which may contribute to slower convergence, as shown in Table 5, or difficulties in optimizing all parameters effectively. This increased complexity may also render the model more susceptible to overfitting, as indicated by the higher variance in validation loss. The comparison between FCN with ResNet50 and ResNet101 underscores the impact of deeper networks. ResNet101, being a deeper model than ResNet50, generally allows for the learning of more complex features. However, the slightly higher training Dice loss in ResNet101 suggests that, while it has the potential to learn more detailed representations, the benefits may diminish, particularly if the dataset is insufficiently large to fully exploit the deeper network’s capacity. Similarly, DeepLabv3 with ResNet50 and ResNet101 shows relatively close performance. The architecture of DeepLabv3, which incorporates atrous convolutions and multi-scale context aggregation, is intended to enhance feature extraction for semantic segmentation. The minor variation in Dice loss suggests that, although ResNet101 offers more layers and potentially improved feature extraction, the advantages are not significantly superior to those of ResNet50. This may indicate that the additional layers in ResNet101 are not fully utilized, or that the model’s complexity poses challenges in training without overfitting. DenseNet demonstrates the highest Dice loss among the models. Although DenseNet’s architecture employs a dense connectivity pattern that encourages feature reuse and facilitates gradient flow during backpropagation, it lacks skip connections between the feature extractor and the expansion part of the network. Consequently, the poorest results can be attributed to this absence of skip connections.

Segmentation performance is illustrated in Figure 3 for individual slices. In the first and last rows, corresponding to the first and last slices, significant differences are observed around the tails and the lower boundary, just above the heart and lungs. In contrast, the second and fourth rows, representing intermediate slices, exhibit improved boundary delineation across all models. The middle slice, depicted in the third row, demonstrates the best segmentation performance among all slices.

Segmentation efficiency can be evaluated based on two primary aspects: internal segmentation quality, measured by the Dice score, and accuracy in boundary delineation. For the assessment of boundary accuracy, the Hausdorff distance metric was employed to compare different models. Figure 5 illustrates that UNet, UNet++, and DeepLabv3 with ResNets demonstrate superior performance in boundary detection. UNet and UNet++ excel in boundary detection due to their architectures, which are optimized for fine-grained segmentation tasks. DeepLabv3 with ResNets achieves precise boundary detection capabilities by leveraging the ASPP block integrated within its architecture. FCNResNet50 emerges as the model with the smallest carbon footprint, indicating its relative efficiency in terms of energy consumption during training. This efficiency may be attributed to the balance between model complexity and the depth of the ResNet50 backbone, which is adequate to perform well without excessive computational demand. Similarly, DeepLabv3 with ResNet50 also exhibits a relatively low carbon footprint, owing to its effective use of ASPP and multi-scale context aggregation. In contrast, UNet++ demonstrates an unexpectedly high carbon footprint despite having fewer parameters than other models like DenseNet. This can be largely attributed to its complex architecture, which includes nested and dense skip connections. In comparison to the study conducted in^6^, all architectures used in this study demonstrate superior results. Although the datasets differ, the influence of hyperparameters and preprocessing steps cannot be overlooked. Additionally , the imaging protocols and techniques used in 2008 remain clinically relevant in many healthcare settings in Norway and globally. Besides, the dataset was acquired using state-of-the-art protocols approved by the regional ethics committee,ensuring its high quality, which still provide critical insights for developing and assessing segmentation models. This study offers a thorough examination of deep learning architectures specifically for breast region segmentation in DCE-MRI images. We explore the latest advancements in deep learning techniques and assess their applicability and performance in this crucial task. Future work could further enhance pre- and post-processing methods such as intensity inhomogeneity (IIH) and noise reduction, investigate various loss functions to evaluate additional effective factors influencing breast region segmentation. Furthermore, it can be utilized for breast lesion segmentation for just to focus on the breast anatomy and removing heart, lung and noise in the background. Additionally, more relevant and compatible data can be employed to enhance generalizability of the model and testify its efficiency.

## Conclusion

In this study, a deep learning (DL) pipeline was developed to evaluate performance of seven DL architectures for the segmentation of breast regions in DCE-MRI. A boundary was proposed for delineating breast borders, and manual annotation was carried out to provide accurate ground truth data. The efficiency of each architecture was assessed in terms of model training loss, training time, inference time, Dice score, boundary detection accuracy, and carbon footprint. The results indicated that UNet++ exhibited the best overall model performance, demonstrating superior accuracy in terms of Dice score. However, UNet showed better prediction accuracy on the validation set. On the other hand, FCNResNet50 emerged as the most efficient model concerning training time and carbon footprint, while UNet achieved the best inference time, making it suitable for real-time applications. These findings underscore the trade-offs between different models, highlighting that each architecture has its strengths and weaknesses depending on the evaluation criteria. For instance, UNet++ provides high segmentation accuracy but requires longer training times, whereas FCNResNet50 is more environmentally sustainable with a lower carbon footprint and quicker training times. This study emphasizes that the choice of model should be based on specific requirements and constraints of the application at hand. Given the increasing importance of sustainability, carbon footprint has become a crucial factor in model selection. Consequently, FCNResNet50 is identified as the most competitive model, balancing excellent performance and efficiency with a moderate inference time.

## Data Availability

The Stavanger dataset analyzed in this study contains sensitive patient information and therefore not publicly available. It will be available upon reasonable request by contacting Endre Grøvik through the institution.

## Code availability

The code for data processing, analyzing and modelling is available on GitHub. To access the code repository, please follow the link on GitHub..

## References

[1] Acciavatti, R. J. et al. Beyond breast density: risk measures for breast cancer in multiple imaging modalities. *Radiology* **306**(3), 222575 (2023). 10.1148/radiol.222575 PMC9968778 36749212

[2] Lew, C. O. et al. A publicly available deep learning model and dataset for segmentation of breast, fibroglandular tissue, and vessels in breast mri. *Scientific reports* **14**(1), 5383 (2024). 38443410 10.1038/s41598-024-54048-2 PMC10915139

[3] Ojala, T., Näppi, J. & Nevalainen, O. Accurate segmentation of the breast region from digitized mammograms. *Computerized medical imaging and graphics* **25**(1), 47–59 (2001). 11120407 10.1016/s0895-6111(00)00036-7

[4] Jiao, H. et al. Deep convolutional neural networks-based automatic breast segmentation and mass detection in dce-mri. *Computational and Mathematical Methods in Medicine* **2020**(1), 2413706 (2020). 32454879 10.1155/2020/2413706 PMC7232735

[5] Ivanovska, T. et al. A deep learning framework for efficient analysis of breast volume and fibroglandular tissue using mr data with strong artifacts. *International journal of computer assisted radiology and surgery* **14**, 1627–1633 (2019). 30838510 10.1007/s11548-019-01928-y

[6] Huo, L. et al. Segmentation of whole breast and fibroglandular tissue using nnu-net in dynamic contrast enhanced mr images. *Magnetic Resonance Imaging* **82**, 31–41 (2021). 34147598 10.1016/j.mri.2021.06.017

[7] Velden, B. H., Janse, M. H., Ragusi, M. A., Loo, C. E. & Gilhuijs, K. G. Volumetric breast density estimation on mri using explainable deep learning regression. *Scientific reports* **10**(1), 18095 (2020). 33093572 10.1038/s41598-020-75167-6 PMC7581772

[8] Yue, W. et al. Deep learning-based automatic segmentation for size and volumetric measurement of breast cancer on magnetic resonance imaging. *Frontiers in Oncology* **12**, 984626 (2022). 36033453 10.3389/fonc.2022.984626 PMC9404224

[9] Giannini, V., Vignati, A., Morra, L., Persano, D., Brizzi, D., Carbonaro, L., Bert, A., Sardanelli, F. & Regge, D. A fully automatic algorithm for segmentation of the breasts in dce-mr images. In: 2010 Annual International Conference of the IEEE Engineering in Medicine and Biology, pp. 3146–3149 (2010). IEEE 10.1109/IEMBS.2010.5627191 21096592

[10] Saffari, N. et al. Fully automated breast density segmentation and classification using deep learning. *Diagnostics* **10**(11), 988 (2020). 33238512 10.3390/diagnostics10110988 PMC7700286

[11] Ertaş, G. et al. Breast mr segmentation and lesion detection with cellular neural networks and 3d template matching. *Computers in biology and medicine* **38**(1), 116–126 (2008). 17854795 10.1016/j.compbiomed.2007.08.001

[12] Zhang, Y. et al. Automatic breast and fibroglandular tissue segmentation in breast mri using deep learning by a fully-convolutional residual neural network u-net. *Academic radiology* **26**(11), 1526–1535 (2019). 30713130 10.1016/j.acra.2019.01.012 PMC6669125

[13] Piantadosi, G., Sansone, M. & Sansone, C. Breast segmentation in mri via u-net deep convolutional neural networks. In: 2018 24th International Conference on Pattern Recognition (ICPR), pp. 3917–3922 (2018). IEEE

[14] Xu, X., Fu, L., Chen, Y., Larsson, R., Zhang, D., Suo, S., Hua, J. & Zhao, J. Breast region segmentation being convolutional neural network in dynamic contrast enhanced mri. In: 2018 40th Annual International Conference of the IEEE Engineering in Medicine and Biology Society (EMBC), pp. 750–753 (2018). IEEE 10.1109/EMBC.2018.8512422 30440504

[15] Yao, J., Chen, J. & Chow, C. Breast tumor analysis in dynamic contrast enhanced mri using texture features and wavelet transform. *IEEE Journal of selected topics in signal processing* **3**(1), 94–100 (2009).

[16] Nie, K. et al. Development of a quantitative method for analysis of breast density based on three-dimensional breast mri. *Medical physics* **35**(12), 5253–5262 (2008). 19175084 10.1118/1.3002306 PMC2673600

[17] Sui, D., Huang, Z., Song, X., Zhang, Y., Wang, Y. & Zhang, L. Breast regions segmentation based on u-net++ from dce-mri image sequences. In: Journal of Physics: Conference Series, vol. 1748, p. 042058 (2021). IOP Publishing

[18] Radak, M., Lafta, H. Y. & Fallahi, H. Machine learning and deep learning techniques for breast cancer diagnosis and classification: a comprehensive review of medical imaging studies. *Journal of Cancer Research and Clinical Oncology* **149**(12), 10473–10491 (2023). 37278831 10.1007/s00432-023-04956-z PMC11796781

[19] Ronneberger, O., Fischer, P. & Brox, T. U-net: Convolutional networks for biomedical image segmentation. In: Medical Image Computing and Computer-assisted intervention–MICCAI 2015: 18th International Conference, Munich, Germany, October 5-9, 2015, Proceedings, Part III 18, pp. 234–241 (2015). Springer

[20] Zhou, Z., Rahman Siddiquee, M.M., Tajbakhsh, N. & Liang, J. Unet++: A nested u-net architecture for medical image segmentation. In: Deep Learning in Medical Image Analysis and Multimodal Learning for Clinical Decision Support: 4th International Workshop, DLMIA 2018, and 8th International Workshop, ML-CDS 2018, Held in Conjunction with MICCAI 2018, Granada, Spain, September 20, 2018, Proceedings 4, pp. 3–11 (2018). Springer 10.1007/978-3-030-00889-5_1 PMC7329239 32613207

[21] Huang, G., Liu, Z., Van Der Maaten, L. & Weinberger, K.Q. Densely connected convolutional networks. In: Proceedings of the IEEE Conference on Computer Vision and Pattern Recognition, pp. 4700–4708 (2017).

[22] Long, J., Shelhamer, E. & Darrell, T. Fully convolutional networks for semantic segmentation. In: Proceedings of the IEEE Conference on Computer Vision and Pattern Recognition, pp. 3431–3440 (2015). 10.1109/TPAMI.2016.2572683 27244717

[23] He, K., Zhang, X., Ren, S. & Sun, J. Deep residual learning for image recognition. In: Proceedings of the IEEE Conference on Computer Vision and Pattern Recognition, pp. 770–778 (2016).

[24] Chen, L.-C. Rethinking atrous convolution for semantic image segmentation. arXiv preprint arXiv:1706.05587 (2017).

[25] Dice, L. R. Measures of the amount of ecologic association between species. *Ecology* **26**(3), 297–302 (1945).

[26] Kohavi, R. A study of cross-validation and bootstrap for accuracy estimation and model selection. Morgan Kaufman Publishing (1995).

[27] Strubell, E., Ganesh, A. & McCallum, A. Energy and policy considerations for modern deep learning research. *In: Proceedings of the AAAI Conference on Artificial Intelligence* **34**, 13693–13696 (2020).

[28] IEA: Global Energy & Status Report 2019. Licence: CC BY 4.0 (2019). https://www.iea.org/reports/global-energy-co2-status-report-2019

[29] Rosado-Toro, J. A. et al. Automated breast segmentation of fat and water mr images using dynamic programming. *Academic radiology* **22**(2), 139–148 (2015). 25572926 10.1016/j.acra.2014.09.015 PMC4366060

[30] Fooladivanda, A., Shokouhi, S. B. & Ahmadinejad, N. Breast-region segmentation in mri using chest region atlas and svm. *Turkish Journal of Electrical Engineering and Computer Sciences* **25**(6), 4575–4592 (2017).

[31] NVIDIA Corporation: GeForce RTX 4090 Graphics Card. https://www.nvidia.com/nb-no/geforce/graphics-cards/40-series/rtx-4090/. Accessed: 2024-09-02 (2024)

[32] Zhong, J., Zhong, Y., Han, M., Yang, T. & Zhang, Q. The impact of ai on carbon emissions: evidence from 66 countries. *Applied Economics* **56**(25), 2975–2989 (2024).

[33] Tamburrini, G. The ai carbon footprint and responsibilities of ai scientists. *Philosophies* **7**(1), 4 (2022).

